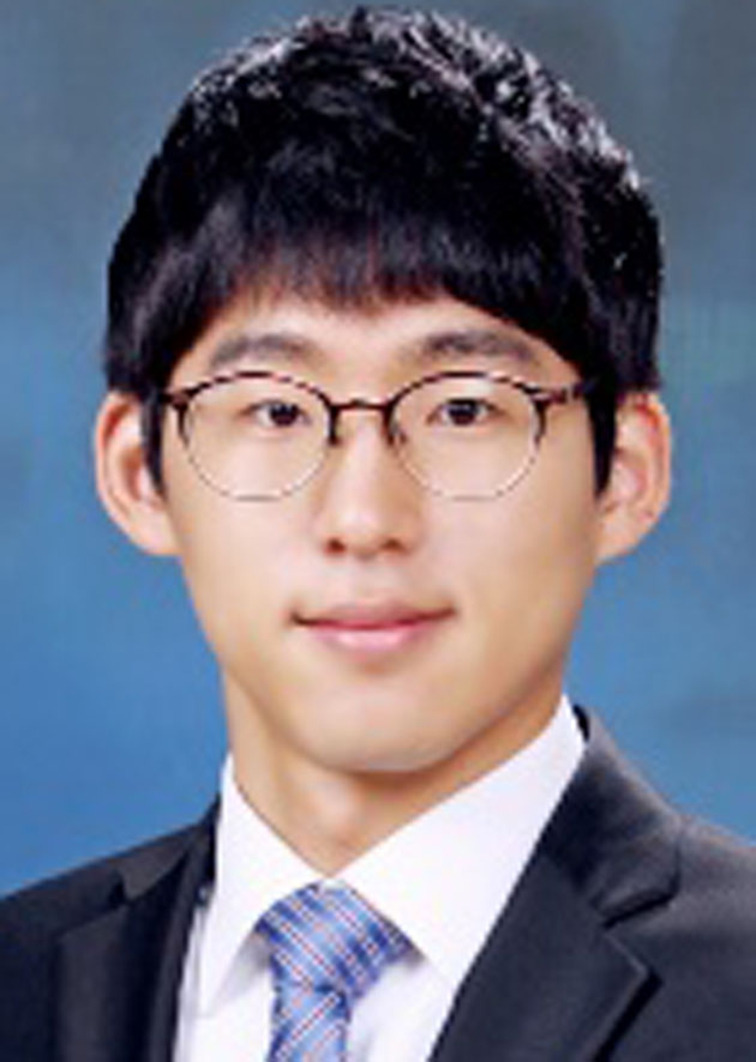# Machine Learning Applications for Chemical Reactions

**DOI:** 10.1002/asia.202200203

**Published:** 2022-05-30

**Authors:** Sanggil Park, Herim Han, Hyungjun Kim, Sunghwan Choi

**Affiliations:** ^1^ Department of Chemistry Incheon Natoinal University and Research Institute of Basic Sciences Incheon 22012 Republic of Korea; ^2^ Digital Bio R&D Center Mediazen Seoul 07789 Republic of Korea; ^3^ Department of Polymer Science and Engineering Dankook University Yongin, Gyeonggi 16890 Republic of Korea; ^4^ Division of National Supercomputing Korea Institute of Science and Technology Information Daejeon 34141 Republic of Korea

**Keywords:** Chemical reaction, Machine Learning, Reaction rate, Reactivity, Retrosynthesis

## Abstract

Machine learning (ML) approaches have enabled rapid and efficient molecular property predictions as well as the design of new novel materials. In addition to great success for molecular problems, ML techniques are applied to various chemical reaction problems that require huge costs to solve with the existing experimental and simulation methods. In this review, starting with basic representations of chemical reactions, we summarized recent achievements of ML studies on two different problems; predicting reaction properties and synthetic routes. The various ML models are used to predict physical properties related to chemical reaction properties (e. g. thermodynamic changes, activation barriers, and reaction rates). Furthermore, the predictions of reactivity, self‐optimization of reaction, and designing retrosynthetic reaction paths are also tackled by ML approaches. Herein we illustrate various ML strategies utilized in the various context of chemical reaction studies.

## Graphical abstract

**Schematic representation** of machine learning(ML) applications for chemical reactions. ML predictions of reaction property and synthetic route are obtained from appropriate selections of ML model and featurization. In this review, recent progresses of ML approaches in chemical reactions are addressed.

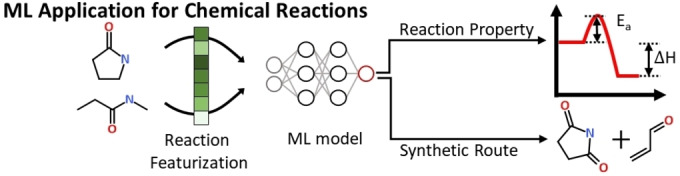

## 1 Introduction

Chemistry is a branch of science that covers the properties of substances and their changes. For the last couple of centuries, experimental and theoretical studies improve our understanding and the predictability of molecular properties and chemical reactions. Recently, a new type of approach, so‐called machine learning (ML), has emerged in many fields of science and engineering.[^1^, ^2^] ML methods are powerful but very general tools to find hidden relationships that are hardly captured by human insight or existing analysis methods.^[3]^ The ML provides useful tools to extend our predictability on many problems at the molecular level.

By utilizing many molecular databases,[^4^, ^5^, ^6^, ^7^, ^8^] numerous interesting ML applications for predicting molecular properties that are originally measured by time‐consuming and expensive experiments or simulations (e. g., toxicity, solubility, and electronic structures) are reported.[^9^, ^10^, ^11^, ^12^, ^13^, ^14^, ^15^] Data‐driven approaches to quantitatively elucidate structure‐property relationships have been studied since the 1980s.[^16^, ^17^, ^18^] The recent ML studies greatly improve the quality and coverage of predictions. Furthermore, ML models are applied to a generative problem which is the design of noble chemical structures for a target property. By the accumulation of large chemical databases with appropriate descriptors, ML applications for various chemical problems are stimulated.

In contrast to the great advances in ML methods to handle chemical compounds, ML studies for chemical reactions, another main subject of chemistry, have been relatively less active due to the lack of data. Recently, with the aid of data‐mining and high‐throughput simulations, chemists can build reaction data libraries beyond lab‐scale or manually constructed reaction databases.[^19^, ^20^] Those stimulate ML applications in chemical reactions. In addition to the size of databases, standardization of chemical reactions are another problem. To describe chemical reactions, it is required to represent not only structural changes of chemical compounds and various chemical agents and conditions (e. g. temperature, solvent, and catalyst). Despite the complexity of chemical reactions, various chemical reaction problems are tackled in the aspect of ML.

In this review, we addressed recent ML applications according to the chemical reaction problems. Those ML studies in terms of various chemical reaction problems. Starting with the illustration of descriptor of chemical reactions and database (Section 2), ML applications to predict physical properties of chemical reactions (Section 3) and synthetic routes (Section 4) were discussed. In Section 3, depending on the target property, physical nature and type of data are largely different. Therefore, we categorized ML studies according to the target properties; thermodynamic quantities accompanying chemical reactions, transition states, and reaction rate & potential energy surface. In Section 4, we addressed three categories of ML studies; predicting reactivity, self‐optimization of chemical reactions, and retrosynthesis.

## 2 Data and Descriptors for Chemical Reactions

### 2.1 Reaction Descriptors

ML models require the input data that is properly transformed into a trainable format. For some digitized data such as images and sounds, no additional transformations are required. But other than digitized data such as natural language demands an appropriate transformation named encoding. For the descriptions of chemical data such as chemical structures and reaction conditions, some available encoding methods have been proposed.

**Figure 1:**
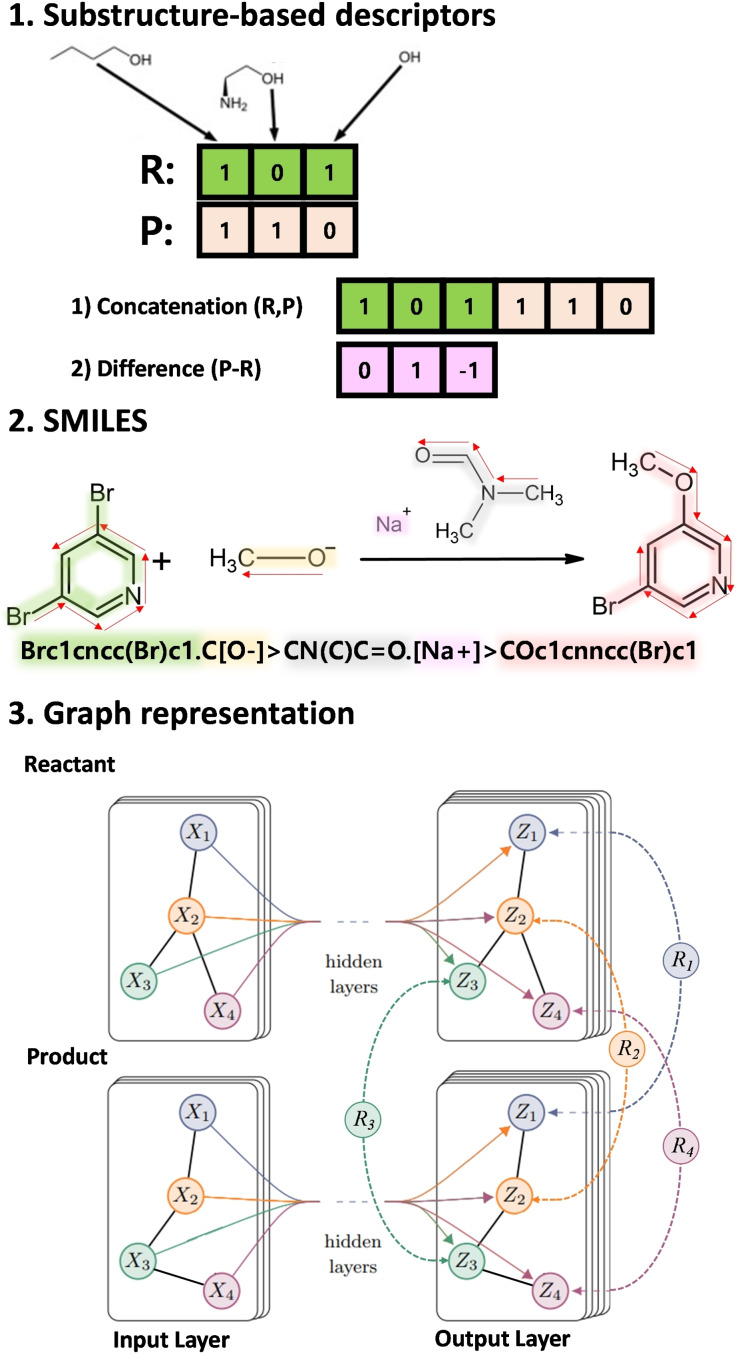
The example of descriptors for chemical reactions. The substructure‐based descriptors represent changes of substructures in the reactant and product. (top) The reaction‐SMILES denotes three parts of reactions: reactants, agents, and products as a single code. (middle) The graphical representation contains node and edge features for atoms and bonds information, respectively. R_1–4_ represent differences or concatenations of node features from reactant and product structures. (bottom)

Descriptors for chemical compounds in the early stage of ML research are designed to reflect the information on substructures such as the number of atoms, bond counts, molecular weight, and fragment counts.^[3]^ For chemical structures, substructure‐based descriptors are widely adopted. Fingerprint and Bag‐of‐bond methods, the two examples of the substructure‐based descriptors, explicitly count the number of predetermined substructures based on atomic connectivities.[^21^, ^22^] Those descriptors explicitly capture substructure patterns in molecules so that molecular properties which are strongly affected by substructures can be effectively learned from those descriptors.^[23]^ Furthermore, chemical reactions can be represented using those substructure‐based descriptors since chemical reactions involve the change of substructures. The reaction features using substructure‐based descriptors are illustrated in the top panel of Figure 1. Since each molecule corresponds to one feature vector, two different ways to represent reactions are available. The first way is to concatenate reactants’ and products’ vectors. It can incorporate the overall features of reactant and product but the dimension of reaction features varies depending on the number of reactant and product molecules. The discordance of feature vector lengths limits its generality. The second way is to calculate the difference of molecular descriptors. It can represent changes of substructures within a fixed length but it does not include information on structures that do not directly change during the reaction.

Although substructure‐based descriptors reflect chemists’ insights well, their applicability is limited due to the lack of whole molecular structure information. It is, in principle, possible to illustrate an overall molecule structure with the substructure‐based descriptors by continuously extending the radius to define substructures/fingerprints. However, typical descriptors consider only a finite number of neighbors to consider substructures because representations of all possible substructures induce enormous lengths for chemical descriptors, which is impractical. The absence of overall chemical structure information influences to represent chemicals as well as chemical reactions. To overcome this limit, the representation that can include whole structural information is highly demanded.

Before ML was introduced in the chemistry field in earnest, a representation called simplified molecular‐input line‐entry system (SMILES) was used to describe chemical compounds as a series of characters.[^24^, ^25^, ^26^] The SMILES translates overall connectivities among atoms in a molecule using the predetermined rules. This method lists the atomic symbol following the backbone of a molecular graph. In the middle panel of Figure 1, red arrows represent the backbone of molecular structures. For branches stemmed from a backbone (like the Br atom in the example), their atomic symbols are written in parenthesis. For the detailed rule of SMILES and its variations, you can refer to the other documents. For its simplicity, many large chemical databases employed SMILES. For instance, GDB‐17 contains 166 billion organic small molecules stored in the format of SMILES representations.[^27^, ^28^] Also, many alternative approaches to encode chemical structures using character sequences have been proposed.[^29^, ^30^] One additional advantage of such character sequences is that the matured ML model natural language processing can be applied seamlessly like a plain natural sentence.[^31^, ^32^, ^33^]

The SMILES can be extended to reaction‐SMILES which represents a chemical reaction. A reaction‐SMILES consists of three parts (reactants, agents, and products) separated by a “>” symbol.^[34]^ In the agent part, reaction conditions such as catalysts and solvents are explicitly included while reactant and product parts contain ordinary SMILES of reactant and product structures, respectively.

SMILES and reaction‐SMILES represent chemical structures and reactions as a sequence of characters so that the arbitrary order among atoms is inevitable. In order to preserve the permutation‐invariance of molecular structures, the graphical representation and the corresponding ML models are introduced. A molecular graph is represented by node and edge features that are derived from atoms and bonds, respectively. Unlike other descriptors, graphical representations consist of heterogeneous quantities (node and edge features) so that a specific type of ML model named graph neural network (GNN) is demanded to preserve graphical nature of data. To build reaction features from a graph representation, difference or concatenation of GNNs outputs, edge or node features, can be used like the substructure‐based features do. The bottom panel of Figure 1 represents that the reaction node features (R_1,2⋯4_), are evaluated from the node features from reactant and product structures. These reaction features can effectively represent changes in molecular structures, but the information for only reactant and product (not reaction condition) are included.

### 2.2 Reaction Database

As we mentioned above, the development of large database allowed the rapid growth of ML applications in chemistry. The database of the early stage for chemical reaction prediction was mostly created based on data published in journals or registered as patents. The United States Patent Trademark Office (USPTO) database, the largest public dataset, was created by extracting more than 3 M reactions from more than 9 M data registered in US patents between 1976 and 2016 using text mining techniques.[^35^, ^36^] This dataset is used for learning various chemical reactions, such as reverse synthesis^[37]^, synthetic analysis^[38]^, reaction classification, and yield prediction.^[39]^ The USPTO has the advantage of having a large amount of data, but there are some incomplete or duplicated reactions. Therefore, by additional filtering, more well‐structured and focused databases are frequently used rather than using the entire set.

Coley's group extracted 15 K organic reactions from the original USPTO database.^[40]^ This database usually named USPTO‐15K is designed to include the rolls of all chemical agents in reactions (e. g. solvent and catalyst) and no duplications. Many ML models to predict products of reactions utilize USPTO‐15K. (Discussed in Section 4.1) Independently of USPTO‐15K, Liu et al. constructed USPTO‐50 K database consisting of 50 K organic reactions with atom‐mapping which is the one‐to‐one map between atoms in reactants and products.^[41]^ The reactions in USPTO‐50 K are selected from the ten predetermined types of the original USPTO reactions. In contrast to USPTO‐50 K, there is another variant of USPTO, USPTO‐380K, with larger number of unclassified reactions.^[42]^ This large database can be used to train less accurate but general ML model for transfer learning. Jin et al. released another USPTO‐based database with 480 K reactions, USPTO‐MIT, without duplicates and chemically incorrect reactions.^[43]^ Pistachio is the USPTO′s extended commercial dataset. While the USPTO dataset includes reactions reported until September 2016, Pistachio covers reactions up to November 2017. Moreover, 13.3 M chemical reactions obtained from ChemDraw sketch data and text‐mined European Patent Office (EPO) patents.[^44^, ^45^]

Besides from the USPTO‐based database, there are reaction datasets from other sources. Reaxys which is only commercially available has 57 M chemical reactions from journals and patents.[^46^, ^47^, ^48^] SPRESI is a manually generated database containing 4.6 M reactions extracted from 700 K references with 170 K patents during the period between 1974–2014.^[49]^ CAS REACTIONS is a database created by the American Chemical Society. It is a dataset containing 144 M single and multi‐step reactions extracted from journals, patents, and papers published from 1840 to the present. Searching in this database can be performed by structures, functional groups, and reaction centers.^[50]^ Current Chemical Reactions (CCR) is a part of the Web of Science provided by the University of Reading. CCR includes 1 M synthesis methods reported in more than 100 organic chemistry journals. This provides detailed reaction information such as reaction conditions, reaction diagrams, and overall reaction pathways.^[51]^

Those databases on chemical reactions were created based on journals or patents, but, recently, Kearnes et al. announced the Open Reaction Database (ORD), for collecting more reaction data. ORD is structured with the schema divided into nine sections: Reaction identifiers, inputs, setup, conditions, notes, observations, workups, outcomes (products and analytics), and provenance. Each schema is flexibly designed to contain diverse chemical reactions. At the initial stage of creation, 2 M reactions extracted from existing databases such as the USPTO were included, and ORD can be expanded by additional registration of reactions from researchers.[^20^, ^52^]

There has been some database for a specific type of reaction. Xu et al. developed a database of asymmetric catalysts for asymmetric hydrogenation of olefins reaction.^[53]^ This dataset was constructed using data collected based on 355 papers during the period between 2000–2020, and the dataset includes four main entity categories (compounds, reaction conditions, reaction performances, and source of publication). This dataset consists of 2,754 olefins and 1,686 catalysts for a total of 12,619 reactions and it enables the hierarchical learning to design the predictive ML model using only olefins and dozens of enantioselectivity data.

From the next section, we will address how reaction descriptors and databases are employed to train ML models on various chemical reaction problems.

## 3 Physical Properties of Chemical Reactions

To obtain deeper insights into chemical reactions, it is essential to predict their observable properties. There are two categories for chemical reaction properties: state‐ and path‐functions. For the case of a state‐function, reaction properties are determined by the initial and final states of a reaction. Enthalpy and entropy changes belong to this category. Another type of property such as reaction barriers is strongly dependent on the reaction coordinates. Here, we introduced some ML approaches to learn both types of reaction properties

### 3.1 Thermodynamic changes

The predictions of atomization energies have received a great deal of attention from the early stage of ML applications in chemistry. By the definition, the atomization energy is an energy change accompanied by breaking all chemical bonds to form isolated atoms. The atomization reactions hardly occur in reality, therefore, atomization energy is more frequently used as a reference energy. By computing the difference of atomization energies for reactants and products, the energy changes during a general chemical reaction can be indirectly evaluated.

For an atomization energy prediction, various GNN models that can systematically learn the chemical environment by considering neighboring nodes and edges have been proposed. (e. g. deep tensor neural network^[54]^, message passing neural network (MPNN^[55]^), and Schnet^[56]^). Those GNNs compute interactions (or messages) among atoms and update node features without loss of permutation‐invariance and size‐extensivity.

To achieve high accuracy and transferability, selections of the model architecture as well as training dataset are important. For molecular property prediction, the QM9 database is a standard database. The QM9 database consists of density functional calculations for ∼134 k small organic molecules. The optimized chemical structures and the corresponding molecular properties are included. All molecules are generated from the enumeration of molecular graphs with up to 9 heavy atoms (C, N, O, and F), which means collecting all possible graphs satisfying the octet rule.[^26^, ^57^] Although density functional calculations provide acceptable accuracy and graph enumeration methods exhaustively span the chemical space, the accuracy and diversity of the QM9 database are still insufficient in the aspect of accuracy and diversity. To supplement the original QM9 database, two different approaches— enhancing accuracy of QM9 database using a higher‐level quantum chemical method and enumerating more molecular structures/configurations— have been reported.

Improving the accuracy of the QM9 database with the G4MP2 method was conducted independently by two different groups; Kim et al. released all results of G4MP2 calculations for QM9 molecules.^[58]^ By comparing G4MP2 results and the B3LYP results from the original QM9, they figured out that there are two types of unwanted geometries in the original QM9; duplicated structures and geometries with multiple molecules (i. e. bimolecular or trimolecular systems). Narayanan et al. also performed the same calculations and compared the result to the experimental data. For the selected 459 molecules, the G4MP2 shows better agreement with the experimental data than density functional calculations do. This improvement of data quality reduces the bias from the database and increases the ML model performances.^[59]^

Enlarging the coverage of molecular databases can contribute to improving the transferability of the trained model. Nakata et al released PubChemQC PM6 dataset which covers 94% of PubChem which is the largest freely accessible molecular database. In addition to the electronically neutral cases, they calculate cationic, anionic, and spin‐flipped electronic states. They validate the accuracy of PM6 calculations by comparing density functional calculations.^[60]^ The aforementioned databases mainly focus on the equilibrium geometries of molecules. The equilibrium geometry is the most frequent pose of a molecule and it is highly relevant to ground‐state properties. Nonetheless, nonequilibrium conformations are also important in various problems, especially in the properties related to dynamics. Smith et al. computed the normal modes of 50 k organic compounds using density functional calculations, which yielded 20 M off‐equilibrium conformations.^[61]^ By training the ML model with nonequilibrium geometries, the accurate molecular energies for both near and far from equilibrium geometries are obtained.^[57]^

**Figure 2:**
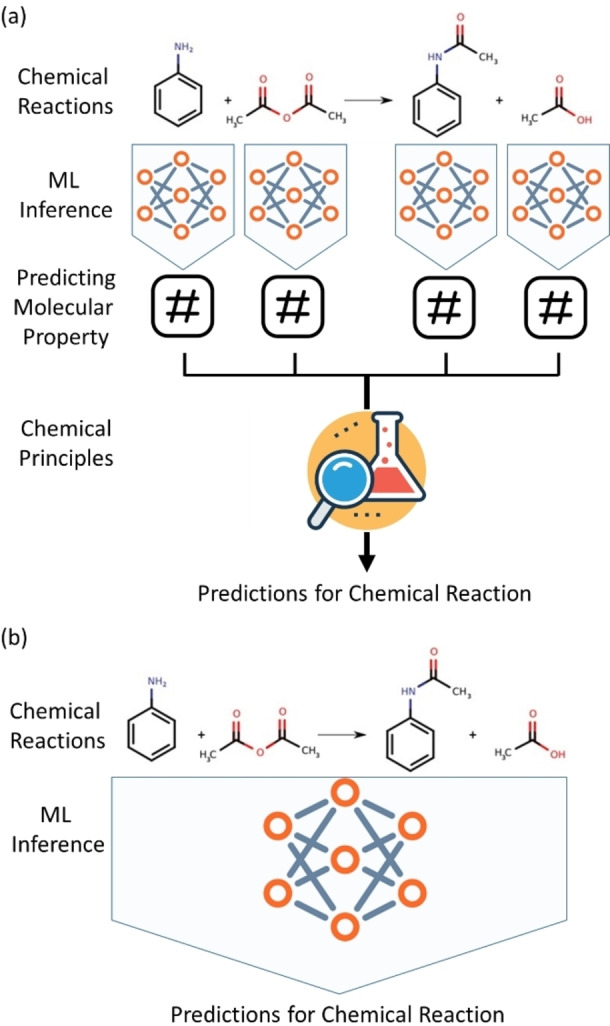
Schematic representations of two different ways to apply a machine learning (ML) model for chemical reaction problems. (a) Reaction properties are computed from the chemical properties predicted by ML. (b) An ML model directly predicts reaction properties from a chemical reaction itself.

Figure 2 represents two ML approaches to predict reaction properties. An ML model predicts molecular properties for reactants and products, and then a reaction property is derived from the predicted molecular properties using physical principles such as the energy conservation law (Figure 2(a)). This approach can predict a reaction property without explicit featurization of reactions. It is applicable only for a state‐function because feature vectors contain the information of individual molecules rather than the reaction itself. However, instead of learning molecular properties, the ML model can directly predict a reaction property based on reaction features without the help of chemical principles. This approach is illustrated in Figure 2(b). In that case, we need to build descriptors for a chemical reaction, and the ML model performs prediction from the reaction features so that a large reaction database is mandatory for training. While acquiring a reaction database is a big huddle in many cases, the second approach is still appealing because it is applicable to predict both state‐ and path‐functions.

**Figure 3:**
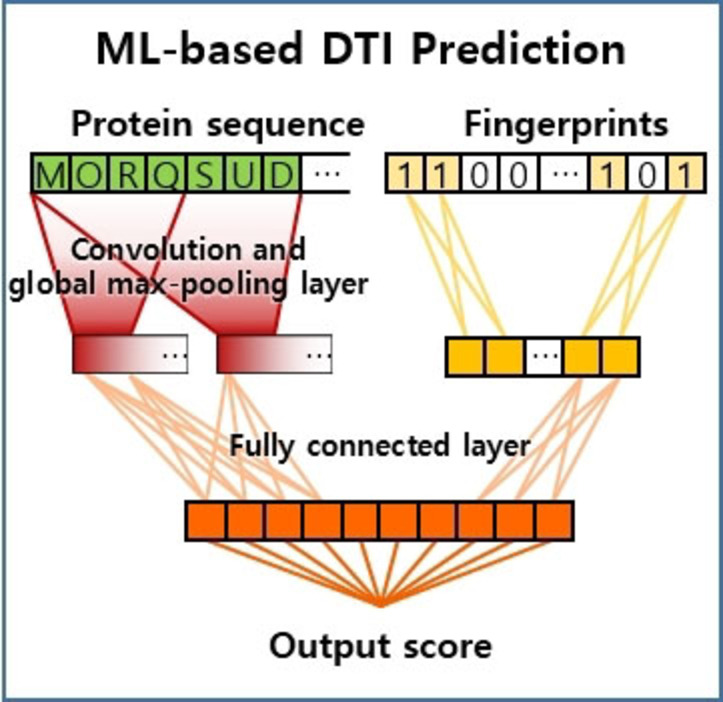
An illustration of the reaction feature construction for drug‐target interaction (DTI). Protein and chemical features are obtained from protein sequence and fingerprint respectively. The machine learning model is employed to find the relationship between the reaction feature and the corresponding DTI value.

A prediction of drug‐target interaction (DTI), the metric to quantify the interaction strength between a target protein and a ligand molecule, is a primary example of the second approach. Although DTI does not involve bond formation or breaking, DTI values vary depending on the combination of proteins and ligands. The estimation of DTI is frequently used to examine the effectiveness of drug candidates.[^62^, ^63^] Estimating strengths of DTI demands expensive experiments or simulation ways. Instead of those heavy methods, ML approaches can make a rapid prediction of DTI which contributes to the acceleration of drug discovery.[^33^, ^34^, ^64^, ^65^] It is quite difficult to obtain reasonably accurate reference energy of a protein due to its huge size and complex structures. Instead of using atomization energies, an ML model takes advantage of reaction features to estimate DTI values. Figure 3 illustrates the process to predict DTI values using ML models. The ML model calculates an output score (DTI) from the reaction feature vector (red vectors) which is derived from the protein sequence (green vectors) and fingerprints of a drug molecule (yellow vectors).

### 3.2 Transition states

A transition state information is essential to determine a chemical rate of reaction, but its identification in an experimental way is a highly challenging task due to its short lifetime. Quantum chemical methods can obtain the structure and properties of the transition state.^[66]^ Those methods are powerful tools to elucidate a reaction profile, however, they are not attractive solutions when the overall kinetic rate is affected by dozens of chemical reactions. Also, the identification of transition states in a computational manner is not well automated, unlike equilibrium geometries. Thus, a huge amount of computational resources as well as human labor are required to complete reaction profiles.

To estimate a large number of reaction barriers, group additivity models are frequently employed.[^67^, ^68^] These methods utilize prior knowledge on reaction barriers for some specific reaction types, so‐called reaction templates. For the given reactions, the group additivity models find an appropriate reaction template and adjust the predetermined reaction barrier considering the chemical environment. This approach is widely employed to elucidate the reactions mechanisms of combustion and explosion. RMG‐Py developed by Green and coworkers includes a large number of reaction templates from simulations/experiments and provides various tools to estimate the kinetics of chemical reactions.^[69]^ Despite several matured theories and accumulated data, the accuracies of group additivity methods are insufficient in many cases.

The transition state database covering general chemical reactions is not easily achievable due to the vastness of reaction space. Therefore, ML applications to predict barrier heights that share the same mechanism are reported. Singh et al. constructed and trained neural network models to predict the performance of heterogeneous catalysts using hand‐craft features that are closely related to the mechanism of catalytic reactions (e. g. a coordination number of the metal atom and identity of the adsorbate).^[70]^ For training, 50% randomly sampled reactions from the entire database containing 249 reactions were used. The trained neural network model predicted activation energies within 0.22 eV error on average which overwhelms the accuracy of Brønsted–Evans–Polanyi relations.

ML models for a specific reaction type can be successfully trained with a small amount of data. This approach can be a practical solution for transition state problems where numerous databases are available on a specific type of reaction. However, it is not transferable to the reactions having different mechanisms.

In order to apply ML models to predict barrier height prediction for general organic chemical reactions, the ML models need to be trained with general chemical features. Choi et al. proposed a general reaction feature based on changes of molecular quantities (thermodynamic quantity, fingerprints, and topological indices) during reactions. Various ML models for the general reactions were trained and validated using reaction data from the RMG database. Although the proposed reaction feature does not include mechanistic information, they achieved a mean absolute error (MAE) of 1.95 kcal/mol for the test set which consists of various types of reactions. However, the reactions belonging to the RMG database are biased toward combustion reactions. Even though the trained ML model and data feature do not include information on reaction types, the recorded performance might not be preserved in other chemical reaction databases.

Green and coworkers constructed a reaction database covering the reaction space more fairly and released activation barriers and molecular structures based on density functional calculations.^[71]^

The reactant and product molecules were sampled from the GDB‐7 database and the corresponding transition states were computed from the single‐ended growing string method which is one of the automated potential energy surface exploration methods. This reaction database contains ∼12 K chemical reactions. Despite the limited size of the reaction database, ∼12 K high quality and regularized chemical reaction data provide the opportunity to study the barrier heights of organic reactions.

In the following work, Green and coworkers proposed the ML model consisting of directed MPNN^[55]^ layers and feed‐forward layers to predict barrier heights of the database released by themselves. The directed message passing layers calculate node features of both reactant and product as usual MPNN^[55]^ layers and subtract reactant's node feature from the product's one. (See the bottom panel of Figure 1) By summing up all node differences, the reaction feature that includes the overall changes due to the reaction can be constructed. The conventional feedforward layers predict barrier heights from the reaction feature. This approach achieved an MAE of 1.7 kcal/mol for a test set.^[71]^

Furthermore, the simulation reaction database is employed to predict geometries of transition states. Since the absolute atomic position is not chemically meaningful, the interatomic distances which are translationally and rotationally invariant are predicted. However, interatomic distances are overcomplete to represent chemical structures. To avoid interatomic distances that are not allowed by mathematical conditions, Green and coworkers built the model that predicts the weight and initial distance matrices rather than predicts the distance matrix directly. The obtained weights and the initial distance matrix are used for the nonlinear optimization of atomic positions. The predicted initial distance matrix does not need to satisfy the conditions fully. Nonetheless, a final distance matrix from the nonlinear optimization is always forced to satisfy the condition because it is constructed from the positions that minimize deviation to the initial distance matrix. For each isomerization reaction, 71% of transition state geometry results in successive convergence of further optimization calculations, which means the ML results are good enough to be initial guess structures of quantum chemical calculations.[^72^, ^73^, ^74^]

### 3.3 Predicting reaction rate and potential energy surface

A reaction barrier elucidates the temperature dependence of reaction rate, but it is insufficient to determine a reaction rate. Strictly speaking, a full understanding of reaction rate requires analysis of all possible reactive trajectories. However, in practice, transition state theory which only considers fixed geometries have been widely adopted because of its simplicity and high accuracy.[^75^, ^76^] In transition state theory, a reaction rate constant, k, is determined by differences of free energy between a reactant and transition state, and a quantum tunneling factor, κ.[^77^, ^78^] The problem is that κ is not simply obtained from the fixed geometry, unlike free energy changes.[^79^, ^80^, ^81^, ^82^]

To obtain accurate κ from an existing database, the Gaussian process model was trained and validated with actual experimental data.^[83]^ The trained Gaussian process model predicts the scaling factor of the existing κ from a traditional transition state theory. In order to extend the coverage of ML predictions, the limited number of experimental tunneling factor data is insufficient. Therefore, instead of real chemical reaction data, Komp and Valleau performed quantum simulations of randomly generated 1D potentials.^[84]^ The generated 1D potentials are designed to represent the energy profiles of molecules along with a hypothetical reaction coordinate. From 1D quantum simulations, they constructed a database for the products of κ and the partition function of reactants which can be computed by integrating transmission coefficient over the entire energy window. A neural network model was trained with the constructed simulation database. The trained model was validated by simulation data as well as realistic chemical reaction data by using experimental reaction barrier information.

Another way to study the kinetics of chemical reactions is to construct a potential energy surface (PES) and generate trajectories on that surface. To construct a PES, an accurate energy function that yields energy of a given geometry needs to be fitted. From the late 1990s, there were attempts to fit flexible neural network functions to obtain a PES before ML techniques were sophisticated.^[85]^ However, those methods tune parameters of the existing pairwise interaction formula, which restricts the flexibility of ML potentials. On the other hand, because a PES consists of energies of various configurations, the rapid inference of ML models described in Section 3.1 can be readily utilized to construct a PES.^[86]^ However, to understand chemical dynamics properly, accurately preserving the symmetry of PES is essential.^[87]^ For this purpose, the permutationally invariant polynomials (PIP) approach is widely used.^[88]^ Instead of directly using interatomic distances, the PIP method constructs symmetrized polynomial vectors and samples configurations on that vector space. Guo and coworkers demonstrated that the neural network is successfully trained with symmetrized polynomial vectors for three‐ and four‐atom systems.[^89^, ^90^] This PIP with a neural network method (so‐called PIP‐NN method) can be also applied to electronically excited systems.^[91]^

A typical quantum chemical simulation based on Born‐Oppenheimer(BO) approximation provides adiabatic energy. However, BO approximation is improper for the geometry in the vicinity of a conical intersection that has degenerated electronic states. To describe mixing two electronic states, a diabatic potential energy matrix consisting of diabatic and coupling potentials is introduced. The elements of the diabatic potential energy matrix are constraint by the group symmetry and permutation‐invariance. PIP‐NN method is accurately trained with satisfying invariance conditions and successfully demonstrated non‐adiabatic photodissociations of H_2_O and NH_3_.

Since the model that learned the PES of a specific molecule cannot be applied to other molecules, it is necessary to repeat the training procedure for each molecule. The PES‐Learn package can automate PES fitting within the ML scheme.^[92]^ It performs geometry sampling and trains neural networks or Gaussian process regressors with the quantum chemical results of sampled configurations. Additionally, it automatically tunes hyperparameters to improve the accuracy of ML models.

## 4 Synthetic Routes

In order to synthesize organic compounds, an appropriate synthetic route needs to be carefully selected. The aforementioned ML models to predict the physical properties of chemical reactions may provide helpful information for chemists to decide the feasibility of a reaction. Nonetheless of such information, only well‐trained chemists can design reaction pathways. To provide a synthetic strategy directly from ML predictions, many ML models are trained with organic chemical reaction databases. Thanks to well‐structured large‐scale organic chemical reaction database, large ML models become trainable.[^19^, ^20^] The first type of ML studies to figure out synthetic routes is to quantify the reactivity and the second category is an optimization of reaction. The last one is retrosynthesis which aims to directly find the starting materials and series of backward reactions from a target material.

The three topics of this section (reactivity prediction, reaction optimization, and retrosynthesis) are closely related and some studies include multiple topics. Many recent retrosynthetic studies include both reaction optimization and reactivity predictions. On the other hand, some reaction optimization studies include optimization of not only reaction conditions but also reactants’ structures. Nonetheless, for ease of explanation, we categorized studies based on their final goals.

### 4.1 Reactivity Prediction

Generally speaking, reactivity indicates the potential for a certain reaction to happen but its strict definition is ambiguous. Therefore, depending on contexts, a reactivity prediction may refer to predicting reaction yield or activation barriers from a given reactant and product molecules. In some other cases, reactivity prediction refers to predicting the molecular structure of a major product.

Many ML studies to predict the performance of catalysts are categorized into the first category. Owing to the diversity of catalysts and ML studies on them, we highlighted a primary example for each catalytic type. For a detailed overview of ML applications in catalyst design, there are some reviews focusing on ML studies for a specific type of catalyst.[^93^, ^94^, ^95^]

For the CO_2_ reduction with the presence of a heterogeneous catalyst, adsorption of CO_2_ on the metal surface is the first and the main bottleneck due to the huge number of possible adsorption sites. In order to explore reactive sites, the ML model is employed. Ulissi et al. applied a neural network model to predict adsorption energies of CO_2_ on various possible configurations of a Ni/Ga catalyst.^[96]^ The approximate adsorption energies from quantum mechanical (QM) simulations of unrelaxed structures are used to train the model. Such a fast prediction enables active site screening for a Ni−Ga catalyst. By this screening, they reduced the number of structural relaxations using QM simulations and efficiently explored many possible configurations to figure out the active site of Ni−Ga catalysts. In this study, catalytic activity is simplified as the adsorption energy on unrelaxed heterogeneous catalysts which is from QM simulations. As we mentioned in the previous section, simulation is one way to accumulate data with manageable resource and time.

On the other hand, QM simulations can be used to construct descriptors to predict an activity of homogeneous catalyst. Hong's group applied an adaptive boosting model to predict the reactivity of hydrogen atom transfer (HAT) reactions.^[97]^ By performing QM calculations for various combinations of catalysts and reactants molecules, a virtual database was constructed. The adaptive boosting model is trained to predict the energy changes of HAT reactions from 56 physical organic descriptors. The physical organic descriptors include local and global features that are derived from QM calculations. Also, they showed that their QM‐based physical organic descriptor can be applied to predict the regioselectivity of radical C−H functionalizations in other literature.^[98]^

In the case of enantio‐selective catalyst reactions, the conventional descriptors described in Section 2.1 cannot be applied because enantiomers share the same structural and electrical properties. To construct an ML model to predict enantio‐selective catalysts, it is essential to build a new descriptor to distinguish mirror‐images in 3D space. There are two different enantio‐selective descriptors.[^99^, ^100^] Average steric occupancy descriptors represent molecules within colored grid points. Each conformer is aligned on an equidistant grid and a value of each grid point increases if the grid point is within van der Waals (vdW) radii. On the other hand, a spherical projection descriptor of molecular stereostructure introduces angular coordinate on a custom sphere and fills the distance between the vdW surface and the sphere on each grid point. In both studies, the authors predicted free energy differences between the transition structures leading to each enantiomer by using their enantio‐selective descriptors. Both descriptors successfully predict the enantio‐selectivity of combinatorically generated catalysts with a typical feed‐forward neural network. The arbitrary factors such as grid setting and the orientation of molecules affect the sensitivity of descriptors, which can be a potential source of bias. In some recent ML studies, ML models that can directly represent 3D point clouds without any arbitrary factors were proposed.^[101]^

In the ML aspect, catalyst performance predictions are more close to conventional molecular property predictions because reactants and products are fixed and the structure of the catalyst is a single variable of the prediction. To predict whether a certain set of compounds react or not, it is essential to collect the negative cases which mean reactions hardly occur. However, most of reaction databases consist of positive data. To overcome this limitation, Carrera et al. generated negative experimental data from the existing positive dataset. They tried to predict whether a given compound reacts with BuNH_2_ or NaCNBH_3_ by training a random forest model with an experimental database. For the negative data, they use two different approaches. The first approach is to generate actual negative data by removing reactive functional groups from the molecules of positive data. This approach is used to generate negative data for test and validation sets. For negative data in the training set, they generated descriptors based on a set of unchanged bonds in positive training data. Using this database, they successfully predict the reactivity of compounds.^[102]^

The result of the aforementioned models is a single scalar value which indicates the size of reactivity but, for the second type of problem, a chemical structure that is non‐trivial in algebraic notations needs to be predicted. To avoid difficulty to predict chemical structures, Nakai and coworkers replaced the original problem with predicting reactive donor and acceptor atoms.^[103]^ Using a QM descriptor, they designed ML to predict most reactive atoms in donor and acceptor and heuristically formed product based on prediction. The QM descriptor is composed of two types of features; Fukui function and orbital information (e. g. orbital energies, MO coefficients from HOMO−2 to LUMO+2, and populations for each atomic orbital). For each atom in donor and acceptor, QM descriptors are evaluated from QM simulations. Using the QM descriptors, they independently trained gradient boosting classifiers for donor and acceptor with a manually built reaction database from an organic chemistry textbook. After the selection of reactive atoms, a final reactive atomic pair is chosen by the ranking model which is trained to select the most reactive atomic pairs from all possible pairs of reactive atoms. The trained model with QM descriptor shows overwhelming performance compared to that with a fingerprint‐based descriptor. In this study, although the chemical structure of a product is not directly derived from ML model, a reaction can be completed by predicting the most reactive atoms for the donor‐acceptor reaction. Although QM descriptor provides accurate results, the computational cost for QM descriptors is a huge obstacle to their application.

To overcome this limitation of QM descriptor, on‐the‐fly generation of QM descriptor was proposed.^[104]^ Using the concatenated feature vector with the generated QM descriptor and graph embedding results using Weisfeiler‐Lehman Network (WLN), a fusion model successfully predicts changes in connectivity due to a chemical reaction. WLN evaluates the difference in graph‐convolutional results and provides atomic features to reflect different connectivities of product and reactant molecules. WLN can be solely utilized to determine reactive atomic pairs.^[105]^ However, they apply global attention to WLN results and concatenate them with QM descriptors. Their QM descriptor is composed of atomic properties (atomic charges, Fukui indices, and atomic shielding constants) and bond properties (bond lengths and bond orders) which are derived from the results of QM simulations. To cut off the computational cost of QM simulation, the author trained a directed MPNN with a large number of pre‐constructed QM descriptors in advance. The trained directed MPNN model predicts QM descriptors from a molecular structure and it is applied to construct the combined atomic feature (QM and WLN) in an on‐the‐fly manner. The pair features are constructed from atomic feature by summing two atomic features. From the pair feature, a fusion model predicts a change of bond orders for each atomic pair. The fusion model was tested to predict chemical structures of products in substitution reactions which are obtained from Pistachio.^[44]^ Without significant increase of prediction time, the fusion model using WLN encoding with on‐the‐fly generated QM descriptors recorded higher accuracy than the results using WLN encoding only.

### 4.2 Self‐Optimization of Reaction

**Figure 4:**
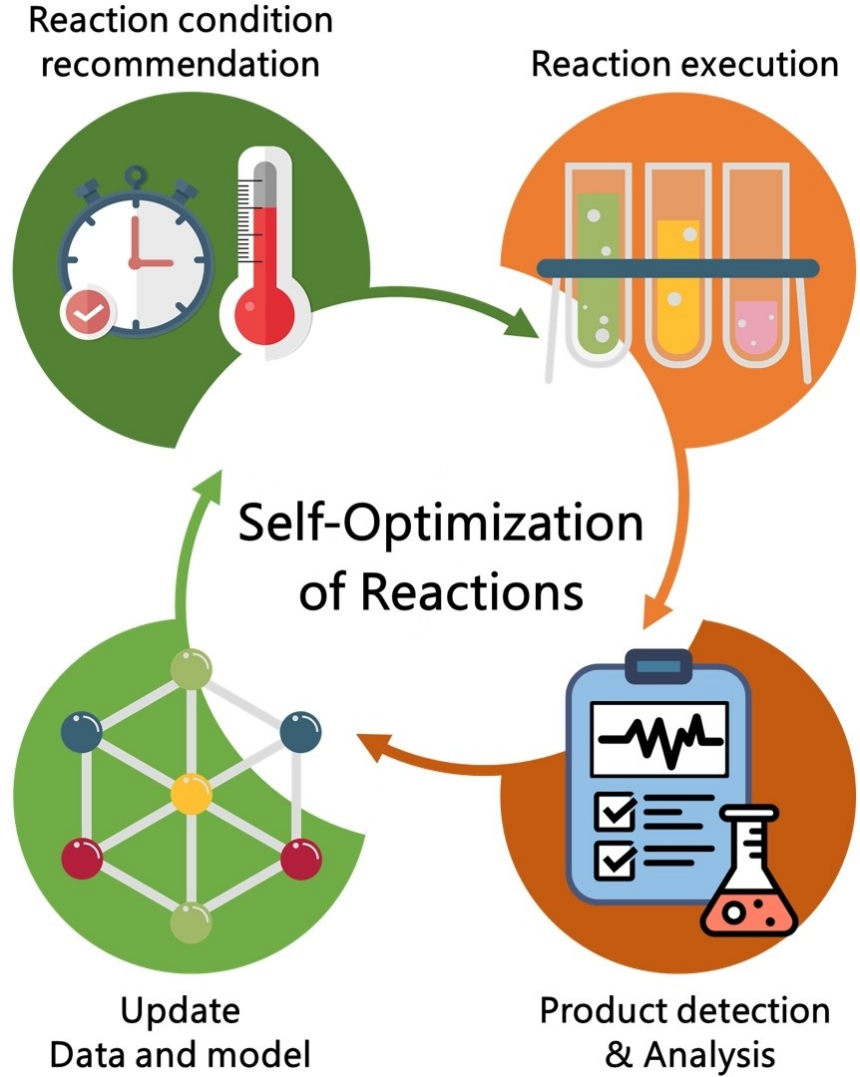
An illustration of self‐optimization of reaction conditions.

Another subject in predicting chemical reactions is the self‐optimization of chemical reaction.^[106]^ This subject is sometimes called by artificial intelligence‐ or machine‐guided optimization because self‐optimization finds the optimal reaction condition by validating ML‐suggested reaction conditions. Conventionally, for reaction optimization, chemists sample candidates of reaction conditions in multidimensional space based on their chemical intuition and results of previous experiments. In the self‐optimization, ML models replaces human intuition in suggesting experimental conditions. These ML approaches are frequently combined with high‐throughput experiment platforms and result in fully automated reaction optimization.[^107^, ^108^] Figure 4 illustrates the iterative process of self‐optimization. Green and red circles indicate the steps performed by computers and high‐throughput experiment platforms, respectively. Without human intuition or labor, the optimal reaction condition can be found by repeating the iterative process.

For exploring vast reaction condition space, many combinations of variables to control mechanical or electrical devices on synthetic platform need to be tested. The relationship of variables to the optimization objective relies on the target reaction and the hardware settings. Therefore, systematic modeling of objective function is hardly available. To overcome this limitation, Bedard et al. reported the application of stable noisy optimization by branch and fit (SNOBFIT) program in a reaction optimization problem.^[109]^ For every step, SNOBFIT algorithm updates subdomains and the corresponding surrogate functions. By optimizing surrogate functions, new sets of reaction conditions are recommended. This method finds optimum without any prior knowledge on reactions.^[110]^

Because SNOBFIT package runs on commercial computing platform, MATLAB, many alternative packages are released. Many newly released packages employ traditional ML algorithms rather than so‐called deep learning methods due to the lack of data and high costs of experimental results. The most widely adopted method is Bayesian optimization which optimally suggests next sampling points by balancing exploitation and exploration.[^112^, ^113^, ^114^] Bayesian optimization packages are available on almost every matured programming communities. Here, we introduce two Python Bayesian optimization packages specialized to the chemical reaction optimization.

First one is TS‐EMO package released by Schweidtmann et al.^[113]^ This package support initial sampling methods and Bayesian optimization with Gaussian processing surrogate models. TS‐EMO package is specialized for optimizing multi‐objectives. The authors reported optimal reaction conditions to maximize the space‐time yield and to minimize E‐factor are found for two different organic reactions.

Another way of Bayesian optimization application was reported by Shields et al.^[114]^ They implemented experimental design via Bayesian optimization (EDBO) and applied it to find the optimal reaction condition as well as functional group of reactants. In order to optimize molecular functional group with reaction conditions, they proposed the new encoding of reactions by concatenating molecular descriptor for each chemical component (reactant, product and solvent) and continuous variables (temperature, reaction time and concentration). For molecular descriptor, they adopt both Mordred descriptor and QM descriptors. Mordred descriptor is a combination of known structural properties such as topological indices and adjacency matrix.^[115]^ It well depicts molecules having different functional groups within 2D or 3D linear algebraic notations. Also, electronic and steric descriptors from QM simulations were also included in the molecular descriptor. Using this molecular descriptor, Gaussian process models with Bayesian optimization maximize reaction yields. In this work, Bayesian optimization is applied by encoding a chemical structure to continuous space. However, in principle, Bayesian optimization can operate with both continuous and discrete variables and there is another package, Gryffin, to support Bayesian optimization with both types of variables.^[116]^ Hase et al. showed that ligands and reaction conditions for Suzuki‐Miyaura reactions are optimally found. Furthermore, it could work with organic solar cells and perovskite materials.

On the other hand, optimization with discrete and continuous variables can be solved through mixed‐integer non‐linear programming (MINLP). Baumgartner et al. reported simultaneous optimization of discrete variables (catalyst types) and continuous variables (temperature, residence time, and catalyst loading) for Suzuki–Miyaura cross‐coupling reactions.^[117]^ The modified MINLP provides the optimal catalyst and reaction condition to maximize the turnover number of catalysts under the maximum yield constraint. By iteratively excluding combinations of discrete variables whose expectation yields are low, the number of candidates is reduced in every step. After the exclusion, continuous variables to minimize uncertainty are selected for each set of candidate discrete variables. By further experiments with the chosen reaction condition, the remained sets of discrete variables are further screened.

In addition to the traditional statistical methods, reaction optimization can be solved by reinforcement learning (RL). The RL is one kind of ML methods to optimize the action policy to maximize or minimize rewards from the environment. RL is frequently adopted to develop artificial intelligence to play games because it finds the optimal routes for an objective with incomplete information.^[118]^ Zhou et al. applied this RL technique to construct the model to find optimal reaction conditions.^[119]^ The action and the reward in the context of RL correspond to the selection of reaction conditions and the yield, the objective of optimization. The policy function is modeled using a recurrent neural network. However, as we explained, such deep learning architecture is hardly trained with a small size of data. To solve this problem, the authors pretrained the recurrent neural network on simulated reactions.

Another example of using deep learning was reported by Gao et al.^[120]^ They trained the neural network model to provide catalysts, solvents, reagents, and temperature from the given reaction descriptors, the difference of Morgan fingerprints. By training the neural network with the large Reaxys database, the model can provide an appropriate reaction condition based on a non‐iterative inference. The trained model provides a reaction condition by learning reaction conditions of similar reactions rather than performing optimization. Nonetheless of this fundamental difference, it was well applied to find a reaction condition in solving a retrosynthetic problem.^[121]^

### 4.3 Retrosynthesis

**Figure 5:**
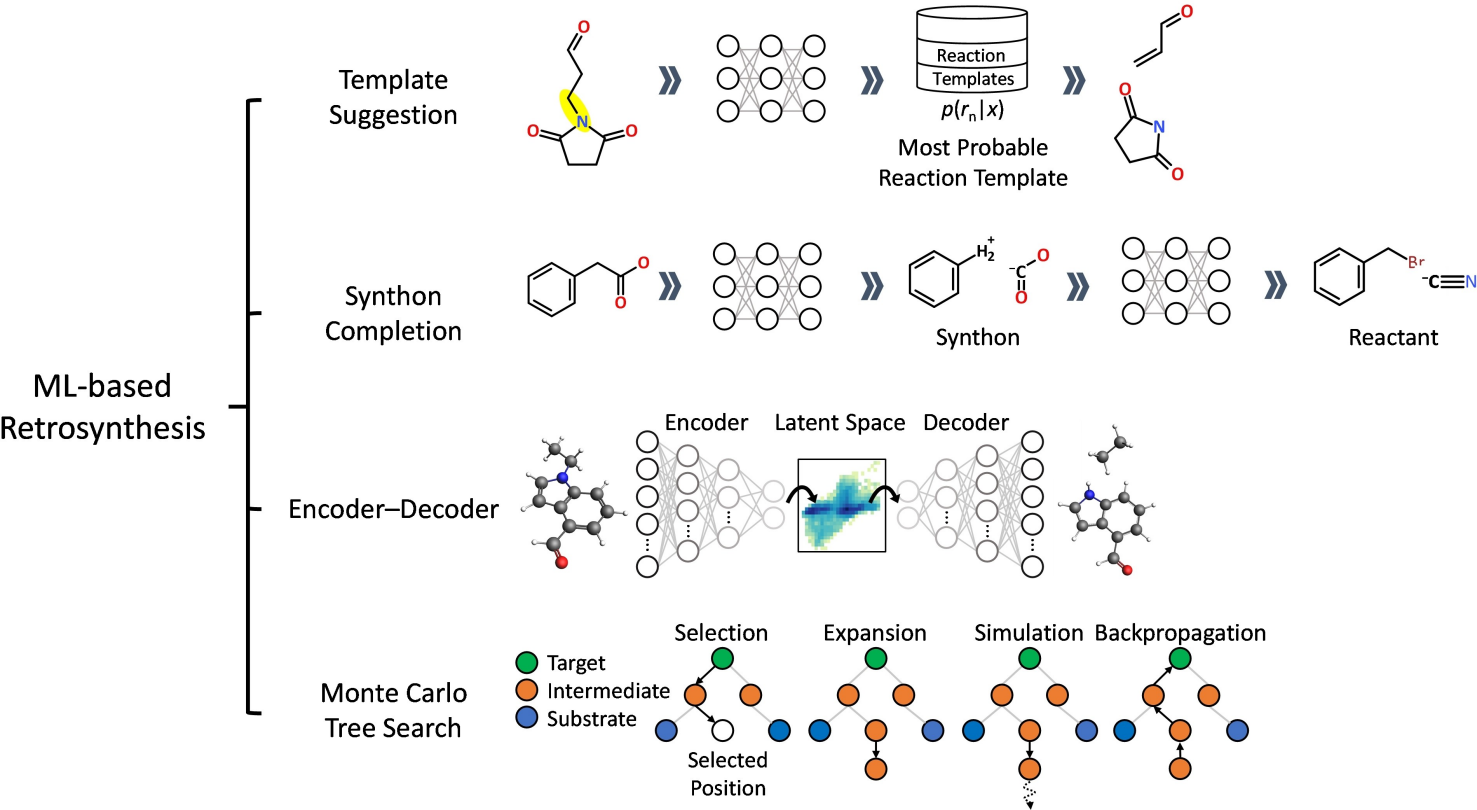
An illustration of categories of ML‐based retrosynthetic predictions discussed in the retrosynthesis part (Section 4.3)

A retrosynthesis also known as synthetic planning is the process of planning synthetic routes for a target product from readily available starting materials. This is one of the challenging problems because the thermodynamic feasibility as well as various reaction environments/conditions need to be considered for an efficient retrosynthetic route. For a long time, well‐trained chemists’ intuition is a unique tool to solve a retrosynthetic problem. After emerging of computers, many computer‐aided retrosynthetic approaches have been proposed.[^123^, ^124^, ^125^, ^126^, ^127^] During the last decade, ML‐based retrosynthetic approaches to improve traditional computer‐aided retrosynthetic approaches or develop completely different approaches have been introduced. In Figure 5, we summarized the ML‐based retrosynthesis approaches covered in this section.

The first category in Figure 5 is a template suggestion based on ML. The template is a type of reactions developed for a traditional retroynthetic approach, rule‐based expert system that performs decision‐making in consultation with accumulated human knowledge. Template suggestion ML models solve retrosynthetic problem with the same way to traditional method in aspect of selecting a reaction template among a predetermined template set based on the given molecular structures. For the prioritization of feasible reaction templates, the expert systems employ a set of heuristic chemical rules. Although the heuristic rules provide plausible reactions in many cases, the finite number of rules can cover only a part of synthetic accessibilities which refer to the feasibility of synthetic routes.

To overcome the shortcoming of the existing expert system, some ML‐based methods to accurately evaluate the feasibilities of reaction templates for a given product have been introduced. Segler and Waller introduced a neural network to select the most probable template from molecular fingerprints.^[128]^ The optimal template among many possible templates was selected by a multiclass classification model. They reported that this classification model can solve forward reaction prediction as well as retrosynthesis problems with overwhelming speed compared to an existing expert system. Similarly, Wei et al proposed a reaction template scoring method based on ML models to solve the forward reaction prediction.^[129]^ The main difference between these two models in the aspect of input features is the way to handle fingerprints of multiple reactant molecules. The model released by Wei et al concatenates the substrate and the secondary reactant. However, the feature for the model released by Segler and Waller is obtained by summing up all fingerprints of reactants.

The aforementioned ML models do not provide the interpretability of their results. To achieve an accurate selection of a reaction template with reasoning, a conditional graph logic network was released.^[130]^ This model decomposes the probability of selecting a specific template into two parts; the probability for choosing a reaction center and the conditional probability of reactants for the given reaction center. This model yields the final probability of the template as well as the probability of choosing a reaction center. The information of reactivity of each substructure can rationale the chosen backward reaction and provide the interpretability of ML inferences.

To enhance the accuracy of the template‐based retrosynthesis model, the ML model using a new type of template was reported. The traditional templates include a reaction center as well as some neighboring structures to represent the required chemical environments. The modern GNN can build atom and bond features including the effect from neighboring or overall structures. Chen and Jung introduced the concept of local templates which represent atom and bond changes without neighboring substructures and LocalRetro model to solve a retrosynthetic problem. This model predicts an atom (or bond) template for each atom (or bond) in a molecule based on atom (or bond) features from the global reactivity attention layer followed by MPNN^[55]^. The model directly predicts reactants from a graphical representation of the product molecule by selecting the most probable local templates among predicted templates on all atoms and bonds.

Existing templates reflect chemists’ insight which can be human bias. Therefore, a generative model to discover new templates from existing reaction data which are free from human bias was proposed.^[131]^ The generative model that is trained with existing chemical reactions generates new chemical reactions which were not reported before. Through the trained generative model with the USPTO database, 31 novel reaction centers and 13 neighborhoods of known reactions are discovered.

Despite many efforts to expand and diversify the reaction templates, template‐based methods have a fundamental limitation that they fail to elucidate the chemical changes outside of preselected reaction templates. As an alternative, template‐free retrosynthesis methods are reported. Since a template‐free method does not rely on the guidance of predefined templates, the results of a template‐free method are exposed to problems of generating chemically nonsensical structures. In order to solve this problem, the concept of synthon completion has been widely adopted. A synthon is a hypothetical (incomplete) structure generated from breaking down a certain bond of product molecule.[^132^, ^133^] Many template‐free ML approaches for a retrosynthetic problem first generate synthons from starting material, and then complete a chemical reaction by modifying synthons based on chemical rules.

Somnath et al. proposed two different models responsible for generating synthons and completing synthons.^[134]^ The first model calculates bond edit scores by encoding an input chemical graph. It generates incomplete structures called synthons. The second model predicts a leaving group from a set of 170 preselected groups. The authors showed that the two models can be jointly or separately trained and a beam search using two trained models successfully finds the best cumulative score of both models.

A synthon completion is also performed by a sequence of graph edits suggested by a generative model rather than a discriminative prediction of leaving group.^[135]^ For that, Shi et al proposed a generative model whose results depend on both latent vectors and synthons’ features. The generative model chooses one of the actions amongst termination, nodes selection, and edge labeling. The node selections determine which atom needs an additional bond and what element will be added. The following edge labeling determines the type of bonding. Such action predictions are repeated until termination is selected. This sequence of actions makes synthon be complete. This sequential editing of a chemical graph is not limited to the retrosynthetic problem. Sacha et al. examined the performance of sequential graph editing on both retrosynthesis and forward synthesis without the help of the ML model to detect reaction centers.^[136]^

The synthon completion methods are useful to handle the incomplete chemical graphs while their applicability is not limited to graph data. Wang et al. proposed the RetroPrime model which consists of two transformers models; the first one splits out the product into a set of synthons (P2S) and the second one generates reactants from the given synthons (S2R). The transformer model is one of the encoder‐decoder models which are a general approach to solve a sequence‐to‐sequence (S2S) problem.

An encoder‐decoder model is powerful to tackle an S2S problem. The changes of SMILES due to reactions can be considered as one of the S2S problems. The first attempt to predict synthetic routes by applying S2S model was done by Nam and Kim.^[137]^ They constructed the attention‐based encoder‐decoder model to predict a forward reaction. In this work, the model directly translates the reactants’ SMILES to the products’ SMILES without the help of the synthon completion. Although they do not utilize additional ML models to complete SMILES, their model achieved reasonable Tanimoto scores for forward reaction problems. Liu et al. further developed this idea to handle retrosynthetic problems.^[41]^ For the retrosynthetic problem of USPTO database, their template‐free model achieved comparable performance with a templated‐based baseline model.

As we mentioned earlier, SMILES cannot satisfy the permutation‐invariance. To solve this, Coley et al. proposed the permutation invariant Graph‐to‐Sequence (G2S) model. The G2S model uses a directed graph attention network (D‐GAT) as an encoder, a variant from MPNN. Encoding the molecule as a permutation invariant graph, this method simplifies data preprocessing and reduces training time. By applying a permutation invariant encoder, the template‐free method can be implemented beyond the limits of the character sequence.^[138]^ To synthesize a complex chemical, multi‐step chemical reactions are frequently required. In principle, the aforementioned methods can be extended to multi‐step reactions, but it is difficult to train a large number of multi‐step reactions within a reasonable computational cost. Monte Carlo tree search (MCTS), a probabilistic way to find the optimal selection on a tree, can explore various multi‐step retrosynthetic routes efficiently. In retrosynthetic problems, each node and branching in a tree represent a chemical structure and backward reaction. The possible chemical reactant structures can be explored by expanding a tree with probabilistic sampling. To obtain the proper solution from MCTS algorithm, it is important to design the expansion and rollout policies. The expansion policy determines how to generate candidates for child nodes and the rollout policy evolves the tree to the terminal node. For the case that only a limited number of actions is allowed at once, the expansion and rollout policies are relatively well defined^[139]^ However, for each intermediate state on a retrosynthetic pathway, an immeasurable number of possible reactions are allowed, therefore, the careful restriction of possible chemical changes without the significant loss of accuracy is highly challenging.

Segler et al. reported the template‐based MCTS application for a retrosynthetic problem combined with three neural networks.^[140]^ In order to guide the expansion of MCTS, the first neural network prioritizes reaction templates, and the second neural network estimates the feasibility of top‐ranked templates. The third neural network is designed for the rollout of MCTS. By utilizing those neural networks, the MCTS algorithm provides reasonable synthetic routes as much as literature routes do. Also, it solves a retrosynthetic problem faster than other heuristic best‐first search algorithms. Schreck et al. combined user‐defined cost metrics to the template‐based MCTS method.^[141]^ Their MCTS algorithm can solve a synthetic problem with minimizing the user‐defined cost metric to consider chemists’ interests such as prices of reactants molecules. On the other hand, the template‐free MCTS method was also proposed.^[41]^ The most distinctive difference compared to the template‐based MCTS is that child nodes are generated from an encoder‐decoder model

For a retrosynthetic problem, the USPTO database is widely adopted as a standard database. The USPTO database includes organic chemical compounds with synthetic routes. The performance and main method of the aforementioned retrosynthetic model are summarized in Table 1. Since the USPTO database includes many duplicated and erroneous reactions so the most widely adopted regularized database is the USPTO‐50 k database which includes 50 k chemical reactions belonging to 10 reaction categories. By re‐regularizing the original USPTO database, a larger database was also released.

**Table 1:** Top‐k accuracy for retrosynthesis prediction on USPTO‐50k database when reaction types are unknown and machine learning technique to be used.

Methods	Top‐n accuracy [%]				Methodology			
	1	3	5	10	Prioritization of templates	Synthon Completion	Encoder‐decoder	Monte Carlo Tree Search
AutoSynRoute^[142]^	43.1	64.6	71.8	78.7			✔	✔
SCROP^[143]^	43.7	60.0	65.2	68.7			✔	
GET^[19]^	44.9	58.8	62.4	65.9			✔	
Tied Transformer^[144]^	47.1	67.2	73.5	78.5			✔	
Graph2SMILES (D‐GAT)^[138]^	51.2	66.3	70.4	73.9			✔	
Graph2SMILES (D‐GCN)^[138]^	52.9	66.5	70.0	72.9			✔	
MEGAN^[136]^	48.1	70.7	78.4	86.1			✔	
G2Gs^[135]^	48.9	67.6	72.5	75.5		✔		
RetroXpert^[145]^	50.4	61.1	62.3	63.4		✔		
GTA^[146]^	51.1	67.6	74.8	81.6			✔	
RetroPrime^[147]^	51.4	70.8	74.0	76.1		✔	✔	
GLN^[130]^	52.5	69.0	75.6	83.7	✔			
Aug. Transformer^[148]^	53.2	–	80.5	85.2			✔	
LocalRetro^[123]^	53.4	77.5	85.9	92.4	✔			
GraphRetro^[134]^	53.7	68.3	72.2	75.5		✔		
Chemformer^[149]^	54.3	–	62.3	63.0			✔	
EBM (Dual‐TB)^[150]^	55.2	74.6	80.5	86.9	✔

## 5 Conclusion

In this review, we summarized recent machine learning (ML) applications for chemical reactions. To apply ML approaches, the proper descriptors, model, and numerous data are demanded. Unlike molecular problems, a large number of quantum chemical results are not accessible for chemical reactions because transition state calculations are relatively less automated and still need massive computing resources. Nonetheless of these difficulties, various theoretical/experimental databases are released and they stimulate various ML applications. Here, we discuss ML studies to predict reaction properties and synthetic routes.

There are two different strategies to predict reaction properties through ML models. The first is to predict molecular properties from a well‐trained ML model and derive reaction properties from chemical principles with the predicted molecular properties. A benefit of this approach is that a large reaction database is not required to train the ML model because the ML model does not explicitly learn chemical reactions. Some path‐independent reaction properties (e. g., enthalpy changes) are successfully predicted by this strategy. However, other path‐dependent properties (e. g., reaction barriers) are not solely determined by properties of reactants and products thus the ML models learning reaction features are demanded. The descriptors for chemical reactions are constructed by expanding molecular descriptors (e. g., SMILES and graph notations). Also, the many models for a chemical reaction are inspired by the models for a molecule.

Predicting synthetic routes is one of the most sought‐after and challenging chemical problems. To tackle this problem, several ML approaches have been proposed. In this review, we categorized them into three subjects; predicting reactivity, self‐optimization of reaction, and retrosynthesis. To predict reactivity from existing reaction data, many ML approaches design models to predict more tangible features like the efficiency of catalyst or reactive atoms and train them using a reaction database. In the problem of self‐optimization, the models that can suggest reaction by reflecting the previous experimental results are proposed. For the retrosynthetic problem, various ML models with or without predetermined reaction types are proposed. The predetermined reaction type, named template, can successively reduce the possibility of chemically absurd reactions but, simultaneously, it limits the capability of ML in human intuitions.

Herein, we highlighted various ML models and their applications in chemical reaction problems. We hope that the addressed techniques to extract the information from reaction data will leverage the realization of potential ML applications in other chemical reactions. Although those various techniques are important in ML applications, a large and high‐quality reaction database is essential to train the ML model, especially for a large and sophisticated ML model. In order to extend the applicability of ML approaches to chemical reaction problems, continuous increase of reaction databases highly demanding. From the authors’ aspect, because of the steady progress of chemical theories and experimental techniques, the cost for reaction data will be lowered continually and it will promote the ML applications for chemical reaction problems that are still not fully understood and predicted by our chemical knowledge.

## Conflict of interest

The authors declare no conflict of interest.

## Biographical Information

*Sanggil Park received his M.S. degree in Chemistry from Incheon National University in 2022 in the group of Prof. Hyungjun Kim His master‘s dissertation topic is the analysis of the physical properties of amorphous polymers used as gas separation membranes using molecular dynamics simulation. He is currently working on rapid and accurate estimation of amorphous polymer materials using machine learning approaches*.

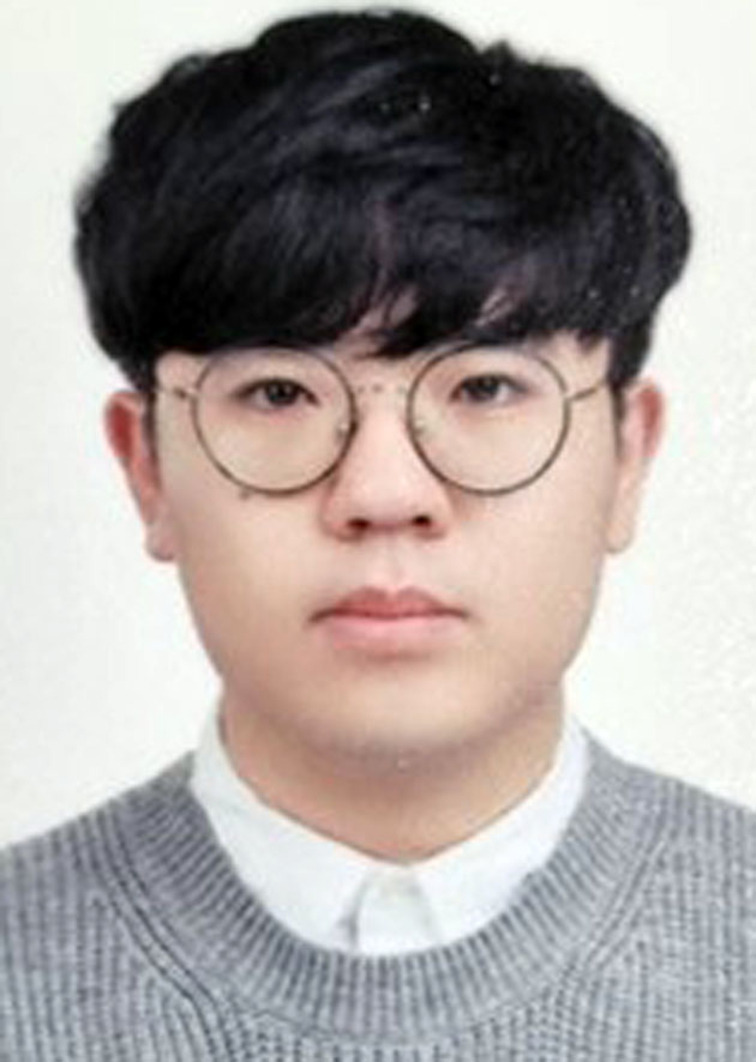

## Biographical Information

*Herim Han received her Ph.D. at Dankook University in the group of Prof. Eung‐Gun Kim in 2022. From July 2020 to January 2021, she was a visiting student researcher at KISTI, where she applied machine learning to the prediction of NMR shifts with the guidance of Dr. Choi. Her Ph.D. dissertation mainly focuses on dielectric effects on thermally activated delayed fluorescence using density functional theory*.

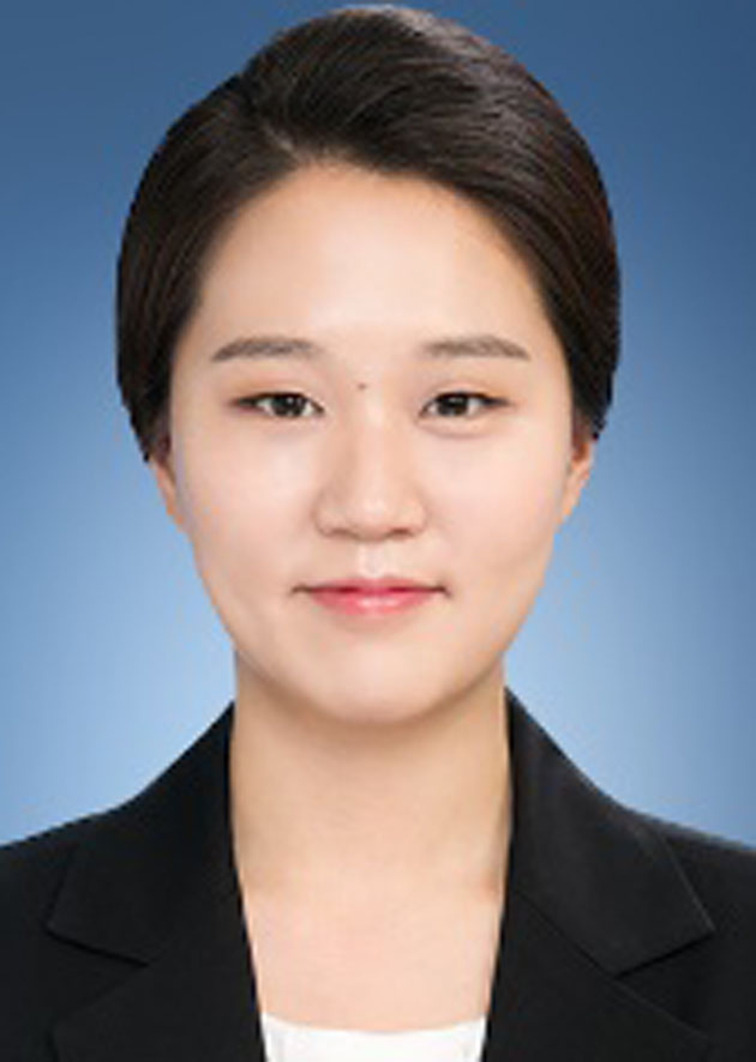

## Biographical Information

*Hyungjun Kim obtained his Ph. D. in physical chemistry at KAIST in 2015. After three years of research experience as a postdoc at University of Michigan, he started his independent research career at Incheon National University in 2018. His research interest is the understanding electronic structures of excited states and applications of machine learning to develop photoactive materials*.

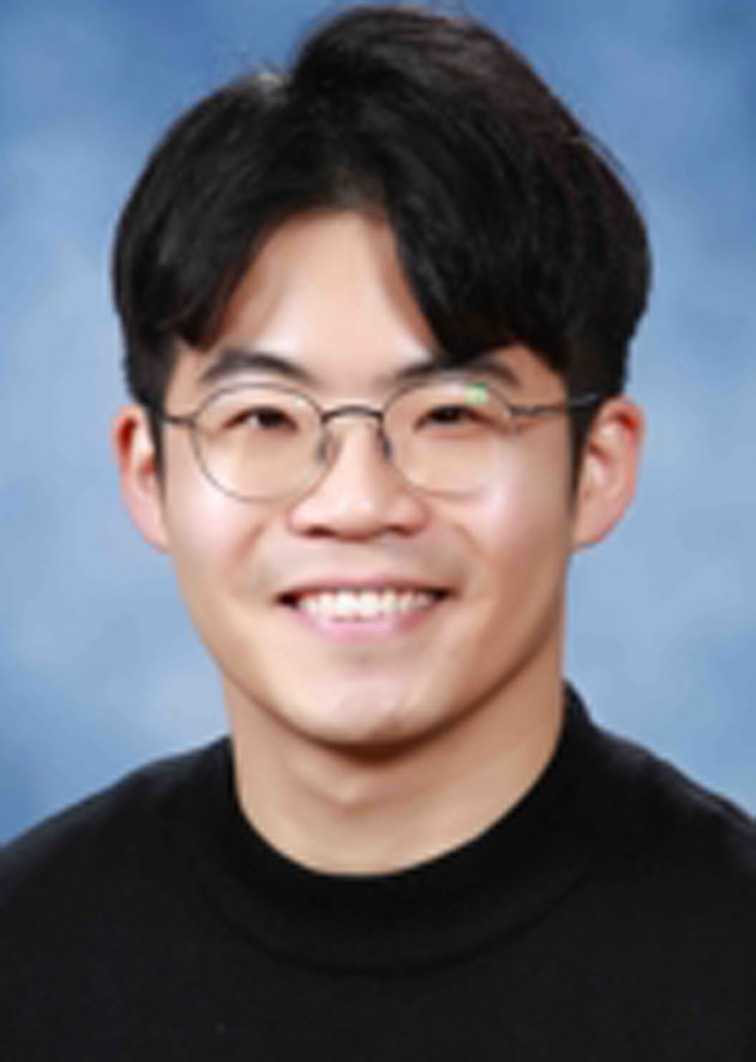

## Biographical Information

*Sunghwan Choi complete his Ph.D in quantum chemistry at KAIST in 2017. After Ph.D, He became a senior researcher at KISTI. His research interest is acceleration of quantum chemical methods using computational or data‐driven techniques*